# Unicompartmental osteoarthritis: High survival rate with a combined mechanical and biological salvage approach as alternative to metal resurfacing: Results at minimum 10 years of follow‐up

**DOI:** 10.1002/ksa.12268

**Published:** 2024-05-20

**Authors:** Luca Solaro, Luca Andriolo, Alessandro Di Martino, Alberto Grassi, Stefano Zaffagnini, Giuseppe Filardo

**Affiliations:** ^1^ Clinica Ortopedica e Traumatologica 2, IRCCS Istituto Ortopedico Rizzoli Bologna Italy; ^2^ Applied and Translational Research (ATR) Center, IRCCS Istituto Ortopedico Rizzoli Bologna Italy; ^3^ Department of Surgery, EOC Service of Orthopaedics and Traumatology Lugano Switzerland; ^4^ Faculty of Biomedical Sciences Università Della Svizzera Italiana Lugano Switzerland

**Keywords:** cartilage, knee, meniscal allograft transplantation, osteoarthritis, osteotomy, unicompartmental

## Abstract

**Purpose:**

The aim of this study was to prospectively evaluate the long‐term clinical results and failure rate of patients treated with complex salvage procedures using a combined mechanical and biological approach to address unicompartmental knee osteoarthritis (OA) and postpone the need for joint replacement.

**Methods:**

Thirty‐nine patients (40.3 ± 10.9 years old) affected by unicompartmental OA (Kellgren–Lawrence 3) in stable joints underwent a personalized surgical treatment depending on the specific requirements of the affected compartment, including high tibial osteotomy, osteochondral scaffold, meniscal scaffold and meniscal allograft transplantation. Patients were evaluated with the International Knee Documentation Committee (IKDC), Visual Analogue Scale (VAS) and Tegner scores before surgery, at 3 years and a minimum of 10 years of follow‐up.

**Results:**

A significant improvement was observed over time in all scores but worsened at the final follow‐up. The IKDC subjective score improved from 46.9 ± 16.2 to 79.8 ± 16.4 at 3 years (*p* < 0.0005) and then decreased to 64.5 ± 21.4 (*p* = 0.001) at 12 years. A similar trend was confirmed for VAS and Tegner scores. Only two patients subsequently underwent knee arthroplasty, and nine more patients were considered clinical failure, for a cumulative surgical and clinical failure rate of 28.2% at the final follow‐up.

**Conclusion:**

A personalized, joint‐preserving, combined mechanical and biological approach, addressing alignment as well as meniscal and cartilage lesions, is safe and effective, providing a clinical benefit and delaying the need for arthroplasty in young patients affected by unicompartmental knee OA. At the final evaluation, the clinical improvement decreased, but more than two‐thirds of the patients still benefited from this treatment at a long‐term follow‐up.

**Level of Evidence:**

Level IV case series.

**Abbreviations**
ACLR: anterior cruciate ligament reconstruction
BMI: body mass index
DFO: distal femoral osteotomy
GLM: General Linear Model
HA: hydroxyapatite
HTO: High tibial osteotomy
IKDC: International Knee Documentation Committee
MAT: meniscal allograft transplantation
OA: osteoarthritis
ROM: range of motion
SD: standard deviation
TKA: total knee arthroplasty
VAS: Visual Analogue Scale

## INTRODUCTION

Osteoarthritis (OA) is a common chronic orthopaedic condition causing a massive impact on the healthcare and socioeconomic systems [19]. In particular, the incidence of knee OA is increasing in young and middle‐aged active patients [37], who represent a very challenging population due to the high functional demands but less effective treatment options with respect to older patients. Despite the overall good results of joint replacements, some controversies remain in the younger population, due to the higher failure and revision rates [5]. A recent registry analysis of almost 50,000 patients who underwent total knee arthroplasty (TKA) documented a relative risk of failure 3.1 higher in patients <50 years old and 1.8 higher in patients 50–65 years old versus older patients [24]. On the other hand, conservative treatments may fail to provide sufficient benefit when addressing severe symptomatic knee in younger patients [32].

During the last decades, several procedures initially developed for young patients, like cartilage treatments and meniscal replacement, were used as salvage procedures in older and OA patients, with the aim to restore articular function and postpone the need for joint replacement procedures [4, 9, 12, 16, 17]. OA is traditionally considered a contraindication [11] for meniscal allograft transplantation (MAT), meniscal scaffold implantation and cartilage procedures [2], but the combination of these procedures with an integrated mechanical and biological approach to address unicompartmental knee OA showed promising results in terms of clinical improvement at short‐term follow‐up [22]. However, since the main outcome of these salvage procedures in young patients is to delay knee replacement, it is important to understand and quantify not only the short‐term clinical improvement but also the rate and time of failure leading to knee arthroplasty over time [8, 36].

The aim of this study was to prospectively evaluate the long‐term clinical results and failure rate of patients treated with complex salvage procedures using a combined mechanical and biological approach to address unicompartmental knee OA. The hypothesis was that the clinical outcome would decrease over time but that patients would still benefit compared to preoperatively, with a high arthroplasty‐free survival rate at a minimum of 10 years of follow‐up.

## MATERIALS AND METHODS

### Patients' selection and treatment

This is a follow‐up study of a series of 43 consecutive patients affected by unicompartmental Kellgren–Lawrence grade 3 OA in stable joints treated with complex salvage procedures, whose results were previously published at a short‐term 3‐year follow‐up [22]. Patients were excluded in case of concomitant ligament lesions, rheumatologic diseases and grade 4 OA.

Patients affected by cartilage lesions in unicompartmental Kellgren–Lawrence 3 OA knees, where previous conservative or surgical treatments had failed, and who were considered too young or refused a prosthetic replacement underwent complex salvage procedures consisting in realignment osteotomy, meniscal scaffold or MAT and/or osteochondral scaffold implantation depending on the specific requirements of the damaged joint compartment. In case of axial deviation, varus alignment was treated with a lateral closing wedge a high tibial osteotomy (HTO) fixed with a staple, whereas valgus alignment was treated with a medial closing wedge distal femoral osteotomy (DFO) fixed with plate and screws. Total and subtotal meniscal deficit were managed with arthroscopic soft‐tissue MAT, while more limited meniscal deficiency with stable anterior and posterior horns and preserved meniscal walls were treated with meniscal scaffold implantation (type‐I collagen meniscal scaffold Menaflex, ReGen Biologics or porous polyurethane scaffold Actifit). Full‐thickness chondral and osteochondral defects of the involved compartment were treated with a type I collagen–hydroxyapatite (HA) three‐layered osteochondral scaffold (Maioregen, Fin‐Ceramica Faenza S.p.A.).

The postoperative rehabilitation protocol depended on the specific treatment combination performed. Patients undergoing realignment osteotomy used an extension brace for a minimum of 30 days to protect the implants. Isometric quadriceps strengthening was encouraged from 24 h after the surgery; passive knee range of motion exercises on a continuous passive motion machine started early in patients not subjected to bony procedures. For osteotomy, MAT and scaffold implantations nonweight‐bearing was prescribed for a minimum of 30 days as a cautionary approach to protect the implants, and then partial and progressive weight‐bearing were gradually allowed. After the initial functional recovery, patients were encouraged to start gentle cycling and swimming exercises to increase strength.

### Patients' characteristics and evaluation

Of the 43 patients previously evaluated at 3‐year follow‐up, four were lost at the final long‐term follow‐up and were, therefore, considered dropouts, while 39 were prospectively evaluated up to a minimum 10‐year follow‐up (mean 12.2 ± 1.3 years).

The final population consisted of 31 men and eight women, with a mean age of 40.3 ± 10.9 years at the time of surgery and a mean body mass index of 25.0 ± 2.3 kg/m^2^. Lesion size in patients affected by full‐thickness cartilage lesions was 4.6 ± 2.1 cm^2^. Thirty‐one patients presented an abnormal alignment, 27 varus and four valgus, and were treated with lateral closing‐wedge HTO and medial closing‐wedge DFO, respectively. Specifically, of the 39 patients evaluated at long‐term follow‐up, 14 patients were treated with osteotomy and cartilage treatment with an osteochondral biomimetic scaffold implant (three of them combined with meniscal substitution), 10 with osteotomy and meniscal scaffold implant, seven with osteotomy and MAT and eight with both cartilage (osteochondral scaffold) and meniscal implants (either meniscal scaffold or MAT). Only two patients were treated surgically for the first time, whereas 37 (94.9%) had undergone previous surgery, often even multiple treatments (27 patients), including 36 meniscectomies, 15 cartilage surgeries (six microfractures, five shavings, three loose body removals and one osteochondral fragment fixation), seven anterior cruciate ligament reconstructions (ACLRs), six tibial plateau fracture treatments, five osteotomies and one medial collateral ligament fixation.

The clinical outcome was prospectively documented preoperatively, at 3 years and at a minimum of 10 years of follow‐up with the International Knee Documentation Committee (IKDC) subjective score (0–100 points), the Visual Analogue Scale (VAS) for pain (10–0 points) and the Tegner score for the activity level (0–10 points). The treatment was considered to have failed if the patient underwent joint replacement in the same knee during the follow‐up period. Besides surgical failures, patients without a clinically significant improvement at final follow‐up were considered clinical failures (10 IKDC subjective score points compared to the basal evaluation, as previously published) [8]. For surgically failed patients, the worst clinical evaluation between the one before the reoperation and the preoperative one was carried forward at the final follow‐up.

The clinical study was approved by the Rizzoli Orthopaedic Institute Ethics Committee (IRB approval protocol n. 0039647), and written informed consent was obtained from all patients.

### Statistical methods

All continuous data were expressed in terms of mean ± SD or mean and 95% confidence intervals. The Repeated Measures General Linear Model with post hoc Sidak correction for multiple comparisons was performed to compare scores at the different follow‐up times. The analysis of variance test was performed to assess the between‐groups differences of continuous and normally distributed and homoscedastic data, the Mann–Whitney test was used otherwise. The Spearman rank Correlation was used to assess the correlation between scores and continuous data. The Kendall *τ*_b_ correlation was used to assess the correlation between scores and ordinal data. Fisher *χ* ^2^ test was performed to investigate the relationships between dichotomous variables. For all tests, *p* < 0.05 was considered significant. No sample size calculations were performed, because of the pilot nature of this study. All statistical analysis was performed using SPSS v.19.0 (IBM Corp.).

## RESULTS

A statistically significant improvement in all scores was observed after treatment but with a decrease between short‐term and final follow‐ups.

**Figure 1:**
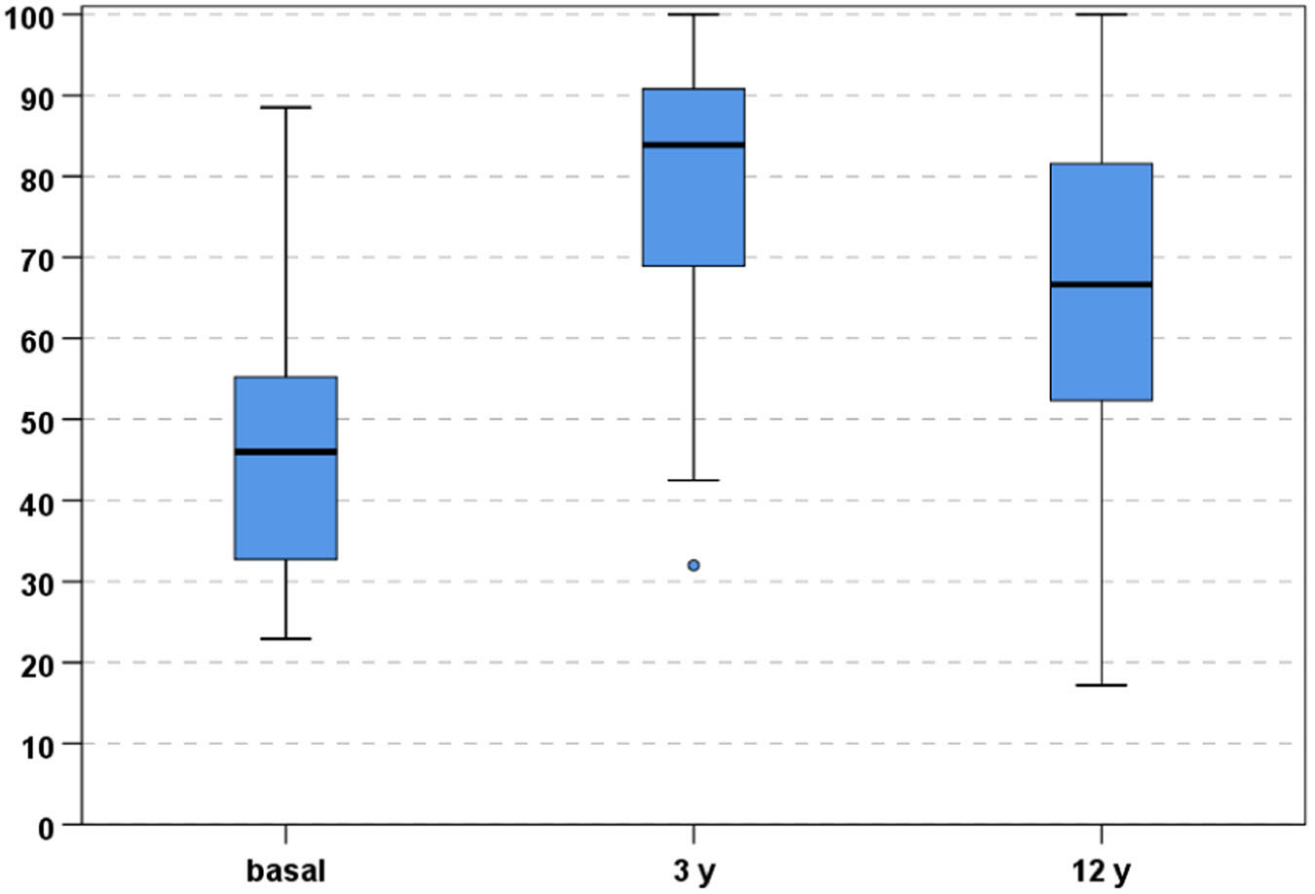
International Knee Documentation Committee subjective score at basal level, 3 years, and final 12‐years follow‐up.

In particular, the IKDC subjective score improved from 46.9 ± 16.4 to 79.8 ± 16.6 at 3 years (*p* < 0.0005), with a subsequent significant worsening reaching 65.0 ± 21.3 at the final evaluation of 12 years (*p* = 0.001), even if the score remained significantly higher compared to the basal level (*p* = 0.002) (Figure 1).

**Figure 2:**
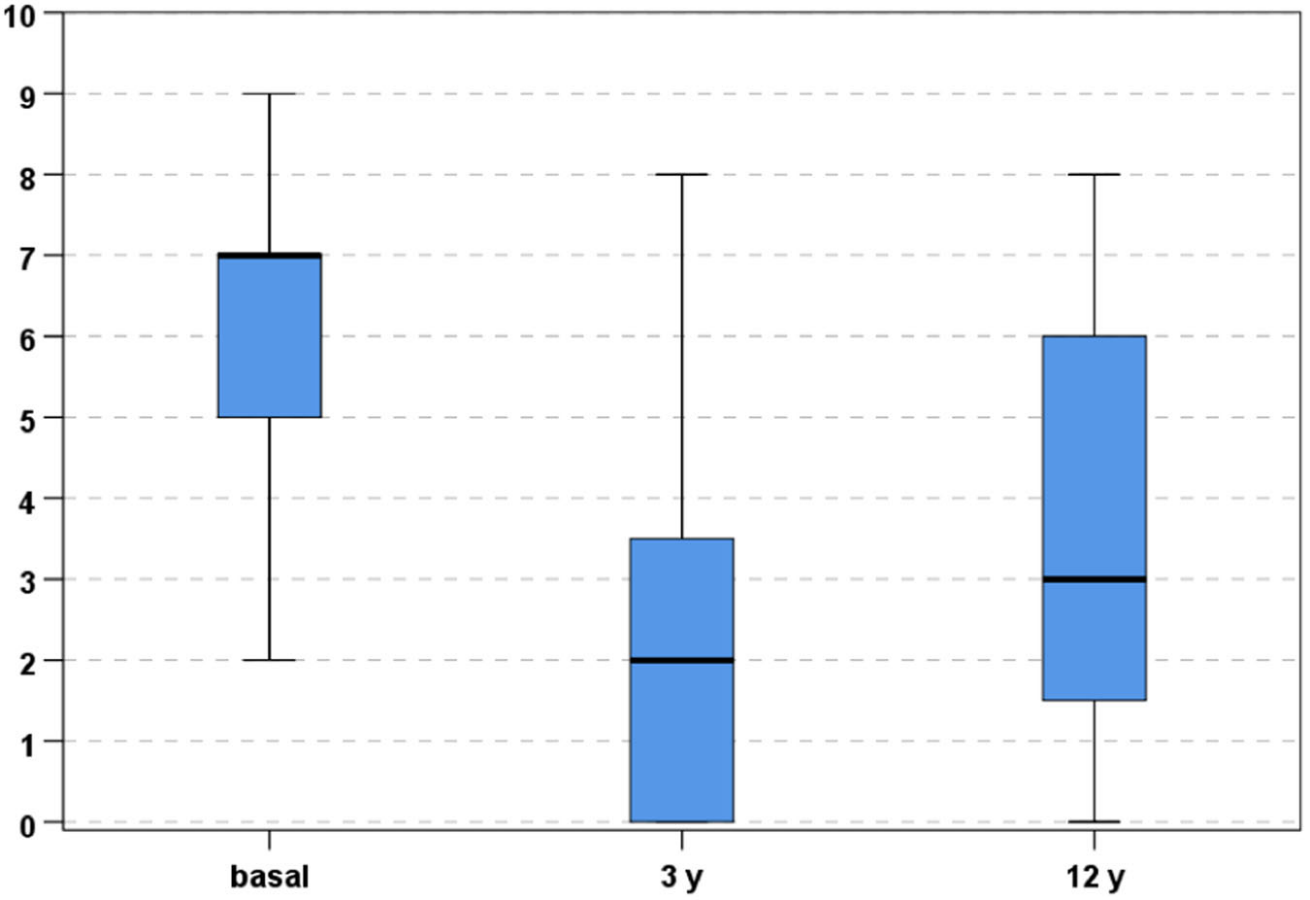
Visual Analogue Scale pain at the basal level, 3 years and final 12‐year follow‐up.

VAS pain score also showed a statistically significant improvement at 3 years (from 6.2 ± 2.0 to 2.2 ± 2.2, *p* < 0.0005), then a statistically significant worsening at the last follow‐up with a score of 3.6 ± 2.8 (*p* = 0.01), but remaining statistically superior compared to the basal evaluation (*p* < 0.0005, Figure 2).

**Figure 3:**
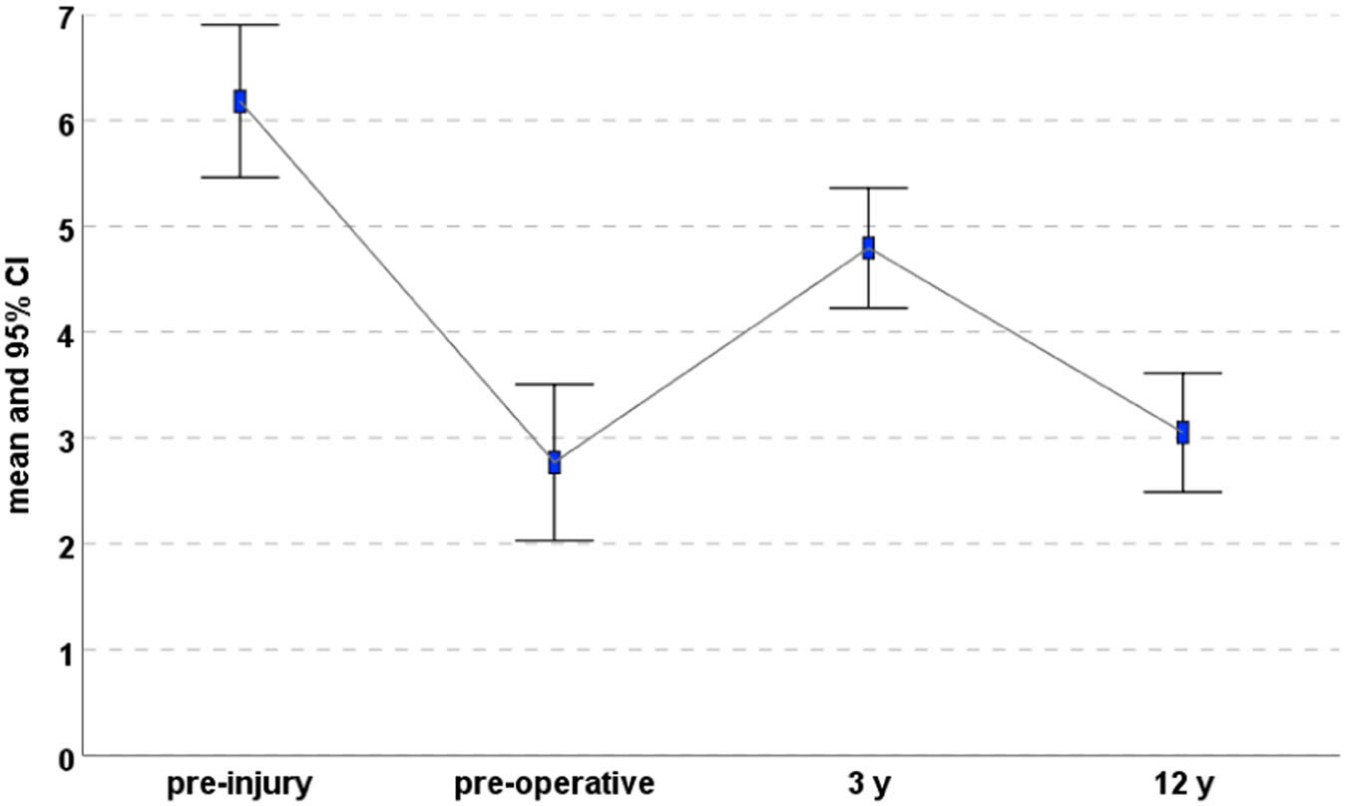
Tegner score at preinjury, preoperative, 3 years and the final 12‐year follow‐up. CI, confidence interval.

The activity level, evaluated with the Tegner score, documented a statistically significant improvement from the preoperative evaluation (2.8 ± 2.3) to the 3‐year follow‐up (4.8 ± 1.7, *p* < 0.0005), but the improvement was not maintained at the final follow‐up (3.0 ± 1.7, *p* = 0.001). Moreover, the activity score remained always lower compared to the preinjury level (6.2 ± 2.2) (*p* < 0.0005) (Figure 3).

**Figure 4:**
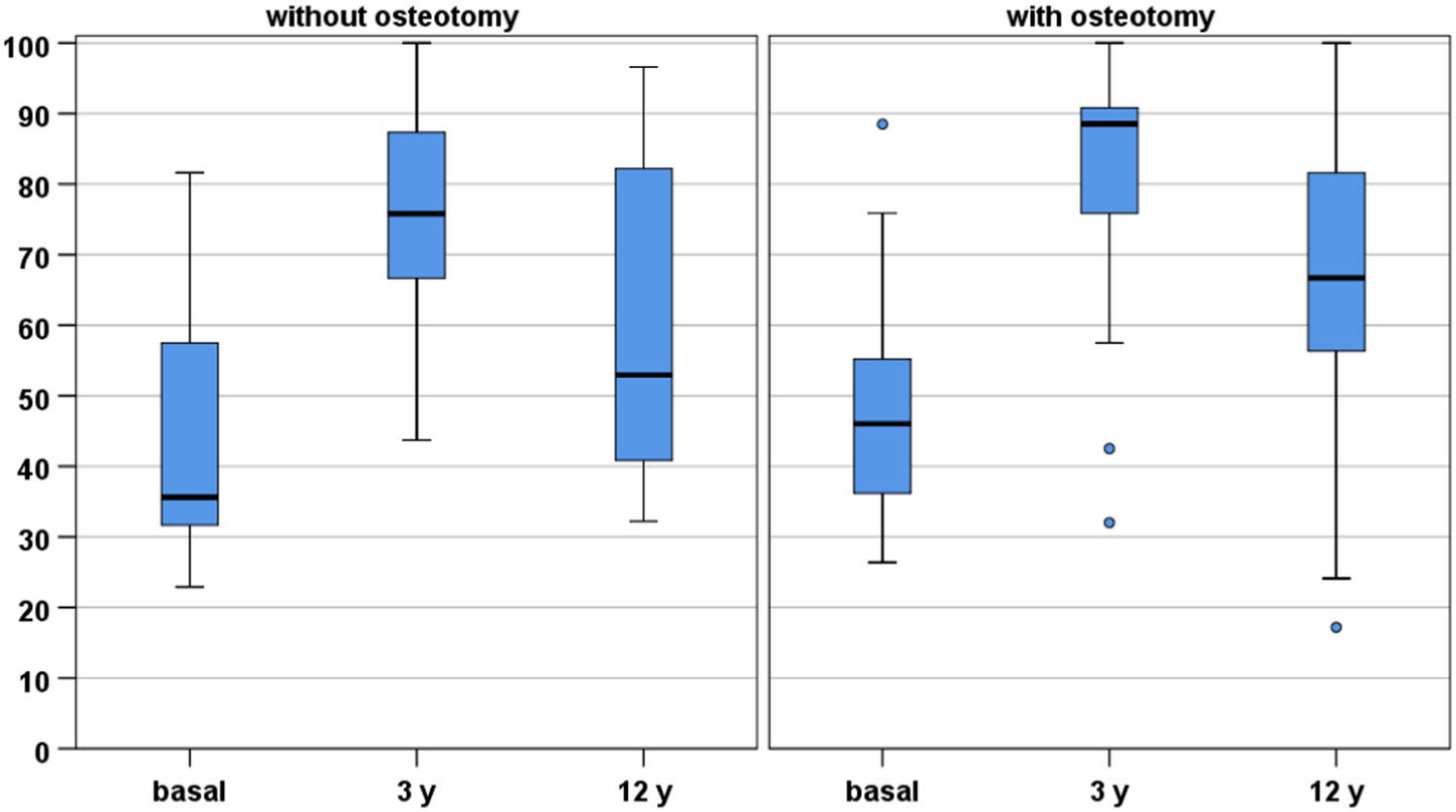
International Knee Documentation Committee subjective score at baseline, 3 years and at the final 12‐year follow‐up in patients with or without realignment osteotomy: no significant difference was found between the groups.

Further analyses were performed to determine the parameters that influenced the clinical outcome at the final follow‐up evaluated with the IKDC subjective score, VAS Pain and Tegner score. Age, gender and BMI did not significantly influence the final outcome. Gender was found to influence the Tegner score at preinjury and 3 years (*p* = 0.044), but not at the final evaluation. The number of combined procedures performed was found to be inversely related to the improvement at the final follow‐up compared to the preoperative level (*p* = 0.020). Patients who required realignment through HTO or DFO showed no differences with respect to those not requiring realignment procedures in terms of clinical score improvement (*p* = n.s.) (Figure 4). Combined meniscal reconstruction with MAT or meniscal scaffold did not influence the long‐term results, as well as previous ACLR (*p* = n.s.).

During the study period, two patients underwent TKA (one patient at 3.7 years, another at 5 years after surgery) and nine more patients failed clinically according to the applied definition, for a total surgical and clinical failure rate of 28.2% at the final follow‐up. No factors were found to be influencing treatment survival, including age, sex, BMI or the presence of combined osteotomy, meniscal reconstruction or osteochondral scaffold implantation.

## DISCUSSION

The main finding of this study is that a personalized combined mechanical and biological approach is effective in providing a long‐term clinical benefit and delaying the need for arthroplasty in young patients affected by unicompartmental OA. At the final evaluation, the clinical improvement decreased, with a cumulative surgical and clinical failure rate of 28.2% 12 years after this salvage procedure.

One of the challenges of treating unicompartmental OA without joint replacement is mainly related to the multifactorial nature of the disease and the articular environment unfavourable towards biological strategies. OA changes are present at many levels in the affected compartment and involve not only the cartilage surface but also the subchondral bone, the meniscus and often a concomitant malalignment [3, 25]. Several procedures have been described to address each of these OA‐related abnormalities, starting with knee realignment via HTO or DFO. For a long time, osteotomies have been the only available surgical option to treat end‐stage unicompartmental OA as an alternative to joint replacement, with satisfactory outcomes in terms of clinical improvement and TKA‐free survival rate [35], without jeopardizing clinical outcomes and survival rate of a subsequent TKA [14]. Consequently, realignment procedures are recognized to play a crucial role in joint‐preservation surgery [15, 29], although they do not address the intra‐articular damaged compartment.

MAT was introduced more than 20 years ago to restore knee function and relieve pain in cases of postmeniscectomy syndrome. The procedure was shown to provide good to excellent results in the general population [23, 27, 33], being effective even in patients older than 50 years old [39]. Moreover, MAT also showed a chondroprotective effect, supporting the rationale of its role in reducing the progression of knee OA [34, 38]. Finally, cartilage regenerative procedures with chondrocyte‐based techniques or osteochondral scaffolds showed promising results, and boundaries were pushed towards more complex cases, including OA knees [1, 6, 7, 9]. Even in this complex condition, a cell‐based approach demonstrated a long‐term improvement with a TKA‐free survival rate of 56% at 15 years follow‐up [2]. For instance, a biomimetic osteochondral scaffold made of type I collagen and HA showed satisfactory outcomes and a clinical success rate of 83.4% for the treatment of focal cartilage defects in the setting of knee OA at 5 years' follow‐up [31], and, more recently, an aragonite osteochondral scaffold showed good to excellent results in a multicenter prospective clinical study at 2 years follow‐up for lesions in mild to moderate knee OA [18].

Correcting the whole joint abnormalities, such as instability, alignment and meniscus deficiency, is mandatory before addressing the articular surface, as every cartilage or meniscal procedure is likely to fail if alignment and stability are not restored [12, 13]. Combined procedures were described in the literature to address these complex clinical scenarios with encouraging results. HTO can be performed together with MAT in varus, meniscus‐deficient knees with satisfactory results [21]. Although with sparse evidence supporting it [10, 25], the combination of HTO and cartilage treatments also showed to provide clinical benefit even in OA joints [28, 30]. Severe meniscal deficiency should be corrected as well when performing articular surface procedures. In fact, both the deleterious effects of meniscal deficit and the positive outcomes offered by meniscal transplantation encourage the combination of MAT and cartilage repair when needed. Previous studies demonstrated the safety, feasibility and efficacy of these combined procedures [26], also in the presence of an OA environment [20].

Based on the rationale of addressing all pathological aspects with personalized combined procedures for treating unicompartmental knee OA, this group of patients was treated with an integrated biological and biomechanical approach, including realignment, meniscal reconstruction and osteochondral treatment. Previously, results published at three years' follow‐up showed encouraging results with a significant clinical improvement and no failures [22]. The present study confirmed the positive outcome with a good survival rate of these salvage procedures and most of the patients avoiding arthroplasty for a minimum of 10 years. However, the clinical results were statistically lower compared to the short‐term follow‐up, documenting a decreased efficacy over time with an overall clinical and surgical failure rate of almost one‐third of the patients. Moreover, the activity level at the last follow‐up was only mildly higher (and not statistically significantly different) compared to the preoperative level. This result is consistent with the low level of activity of patients undergoing cartilage treatments in the case of knee OA, although the older age of the patients at the long‐term follow‐up must be taken into account as a factor influencing the activity level.

Still, despite the lower clinical results, as the main outcome for these patients seeking complex salvage treatments was to postpone metal replacement, it is worth explaining that only 5% of the patients underwent prosthetic implantation at long‐term follow‐up. Overall, these findings suggest the possibility of achieving good clinical and functional results with joint‐preservation combined salvage procedures also in the case of unicompartmental knee OA.

The present study has some limitations, among all, the heterogeneity of the treated patients. However, for these complex patients, a targeted approach is required to address all abnormal conditions and ensure the most favourable outcomes. Thus, a personalized joint‐preserving treatment was planned to tackle both biological and mechanical alterations in this cohort of patients with unicompartmental OA. While the complexity of the combined treatments does not allow the analysis of the specific contribution of each procedure to the clinical outcome, this series documents the safety and potential of pursuing salvage procedures to postpone the need for prosthetic replacement in a real‐world scenario. In this context, another possible objection could be the potential overwhelming influence of osteotomy in determining the clinical outcome, as many patients underwent realignment. In this regard, a subgroup analysis was performed, showing similar benefits in patients not requiring realignment and thus undergoing only intra‐articular procedures. Similarly, the cohort was unbalanced in terms of patient sex, with almost 80% of men; nevertheless, also in this case, a subgroup analysis was performed and no long‐term outcome was found to be influenced by sex. This analysis may be affected by the low numerosity of the series, which is due to the fact that this is a tailored integrated treatment, limiting the possibility of applying this approach in a large and homogeneous group of subjects. Further studies with a properly powered series of patients will help confirm the preliminary findings of this study. Finally, an imaging evaluation at the final follow‐up was not available. Nonetheless, these joints already present OA and a radiological improvement is not expected, while clinical results and failure rate are the most significant outcomes in this cohort of patients evaluated at this long‐term follow‐up.

The high survival rate and the overall positive results of this complex surgical approach suggest that it can be proposed as a salvage treatment option in selected patients willing to delay metal resurfacing. Nonetheless, the results decrease with time and this should be clearly discussed with the patients, together with the long rehabilitation time, the risk of complications due to multiple surgeries and the not negligible risk of clinical failure.

## CONCLUSION

This study showed that a personalized, joint‐preserving, combined mechanical and biological approach, addressing alignment as well as meniscal and cartilage lesions, is safe and effective, providing a clinical benefit and delaying the need for arthroplasty in young patients affected by unicompartmental knee OA. At the final evaluation, the clinical improvement decreased, but more than two‐thirds of the patients still benefited from this treatment at a long‐term follow‐up.

## AUTHOR CONTRIBUTIONS

**Luca Solaro**: Formal analysis and investigation; writing—original draft preparation. **Luca Andriolo**: Formal analysis and investigation; writing—review and editing: **Alessandro Di Martino**: Writing—review and editing. **Alberto Grassi**: Methodology. **Stefano Zaffagnini**: Conceptualization; supervision. **Giuseppe Filardo**: Conceptualization; methodology All authors read and approved the final manuscript.

## CONFLICT OF INTEREST STATEMENT

Stefano Zaffagnini is a consultant surgeon for Smith and Nephew and DePuy Synthes. The remaining authors declare no conflict of interest.

## ETHICS STATEMENT

The study was approved by the Ethics Committee of the IRCCS Istituto Ortopedico Rizzoli (Approval protocol n. 0039647). All the enrolled patients signed the informed consent.

## Data Availability

The Ethics Committee did not authorise the sharing of the raw patients' data. The calculated average values, which protect patients' privacy, are detailed in the manuscript.
